# Trust After Terror: Institutional Trust Among Young Terror Survivors and Their Parents After the 22nd of July Terrorist Attack on Utøya Island, Norway

**DOI:** 10.3389/fpsyg.2019.02819

**Published:** 2019-12-13

**Authors:** Lisa Govasli Nilsen, Siri Thoresen, Tore Wentzel-Larsen, Grete Dyb

**Affiliations:** ^1^Norwegian Centre for Violence and Traumatic Stress Studies, Oslo, Norway; ^2^Center for Child and Adolescent Mental Health, Eastern and Southern Norway, Oslo, Norway; ^3^Institute of Clinical Medicine, Faculty of Medicine, University of Oslo, Oslo, Norway

**Keywords:** institutional trust, terrorism, terror survivors, police, justice system, public institutions

## Abstract

In the aftermath of terrorist attacks and disasters, public institutions play an important role in re-establishing safety and justice. However, little is known about the importance of institutional trust for victims’ potential for healing in the aftermath of mass trauma. This study examines levels of post-terror trust in the police and in the justice system among young survivors from the 2011 Utøya terror attack and their parents. Furthermore, it investigates how institutional trust develops over time among directly affected populations, and whether it is associated with psychological distress. 325 survivors and 463 parents were interviewed face-to-face at wave one (4–5 months post-terror) and 285 survivors and 435 parents at wave two (14–15 months). Levels of institutional trust in victims were compared to general population data from the European Social Survey adjusted for age, gender, and ethnic background. Measures included trust in the police and justice system, post-traumatic stress reactions, anxiety and depression, and quality of life. Trust in the police among survivors and parents was higher than or comparable to trust levels in the general population at wave one, but decreased for survivors and parents at wave two. Trust in the justice system was higher among those directly affected than in the general population, and increased from wave one to wave two. Levels of institutional trust were negatively associated with distress for survivors in both waves and for parents in wave two. Levels of institutional trust were positively associated with perceived quality of life in parents and survivors. Directly affected groups’ institutional trust differed from that of the general population following the terrorist attack, although being directly affected did not necessarily imply weakened institutional trust. This study found trust to be institution specific, however, trust in institutions changed with time, and the passing of time might be an important factor in better understanding whether trust will generalize across institutions or not. Institutional trust was negatively associated with psychological distress. This finding highlights the potential for institutions to create a healing post-disaster environment.

## Introduction

Mass traumas such as terrorist attacks may influence the general public’s trust in institutions (e.g., Wollebæk et al., 2012; Dinesen and Jæger, 2013). Little is known, however, about how institutional trust is affected among those who experience these events first-hand, and how their levels of trust develop as time passes after the disaster. To help fill this knowledge gap, this study examined post-disaster institutional trust among young survivors of the 2011 terrorist attack on Utøya Island in Norway and their parents, as compared to the general population. Additionally, we investigated the temporal development of institutional trust among the directly affected after the terrorist attack, as well as the potential relationship between trust, mental health and wellbeing. The terrorist attack on Utøya island was perpetrated by a single terrorist, who committed a shooting spree targeting young people who were taking part in the Norwegian Labour Youth’s annual summer camp.

Institutional trust is important for society at large; high levels of trust are believed to promote economic growth, increase people’s willingness to engage in communal activities, and contribute to well-functioning democracies (see e.g., Fukuyama, 1995; Putnam, 2000). At the individual level, institutional trust may impact psychological health and wellbeing (see e.g., Hudson, 2006; Lindström and Mohseni, 2009). The concept of trust continues to be debated and competing conceptualizations exist, although a point of consensus is found in the frequent inclusion of a definition of the trustor, the trustee, and the connection between the two (PytlikZillig and Kimbrough, 2016). Moreover, studies of institutional trust are often embedded in examinations of the more overarching concept of social capital. Definitions of social capital vary, but it commonly refers to the ties or linkages between individuals in a network or community, including trust and norms for reciprocity (see e.g., Coleman, 1988; Putnam, 2000). Some argue that social capital is not something that individuals “have,” but rather something that is generated in the social contexts in which individuals find themselves and operate (Newton, 2001). Others perceive social capital to be individually held or perceived [e.g., the concept of cognitive social capital by Wind et al. (2011)]. Trust can be conceived as generalized or particularized, where generalized trust refers to a general belief in a benevolent human nature, while particularized trust is directed toward specific people (Carpiano and Fitterer, 2014). Institutional trust is particularized in the sense that it concerns trust in specific institutions. More specifically, it can refer to the extent to which the trustor expects the trustee (the institution) to carry out its responsibilities to a satisfactory degree (Hudson, 2006). Beyond such instrumental expectations, trust in institutions may also be linked to how individuals perceive their relationship to those institutions. For instance, a study of a group of terror victims and their perceptions of institutions, revealed that they not only expected institutions to carry out their responsibilities, but also to do so in a humane manner, acknowledging the individual needs (Waldman et al., 2011). Furthermore, perceptions of institutions of law and justice, including trust in them, may to some extent derive from experiences of justice. This could include whether one perceives oneself to be identified and acknowledged as a victim, and whether responsibility is placed with the perpetrator (Wenzel et al., 2008).

Major disasters can impact the fabric of society, and a few studies have investigated institutional trust at the societal level in the aftermath of mass trauma. Following terrorist attacks, several studies have found trust to exhibit a “rally effect,” where it increases significantly just after the attack, remaining high for a few months, but returning to pre-attack levels within a fairly short time span of up to a year (Putnam, 2002; Woods, 2011; Wollebæk et al., 2012; Dinesen and Jæger, 2013). Whether the rally effect generalizes across institutions is debated, however, when studying trust in a range of institutions following the 2004 terrorist attack in Madrid, Spain, Dinesen and Jæger (2013) found that even though trust in political, media, judicial, and other institutions generally increased significantly right after the attack, the effect varied across types of institutions, and was more short-lived for some institutions than others.

After terror and other disasters, trust may also vary at subnational levels. A mass trauma represents a strain on any community, but its impact can vary within and across communities (Rumbach et al., 2016), and high-trust and low-trust communities may be affected in different ways (Dussaillant and Guzman, 2014). Communities will differ in terms of the resources they have available to cope with and adapt to a disaster (Bos et al., 2005; Cutter et al., 2008). Often, disasters can be followed by a period of conflict between affected communities and public institutions if the latter are perceived to have failed to uphold their institutional responsibilities (Sauri et al., 2003; Thoresen et al., 2018).

On an individual level, factors such as personal experiences and life course developments may influence levels of trust. Although some believe institutional trust, at least in part, may be learned early in life, others argue that it forms later on, and is based on institutional performance or other factors (Hetherington, 1998; Mishler and Rose, 2001; Hudson, 2006). Oosterhoff et al. (2018) found that discontent with the government in an American youth sample was associated with level of criminal victimization, regardless of demographic characteristics and the socio-political environment at any given time, that is, which party was in government and what their priorities were. These authors therefore argue that discontent with government, including a lack of trust in public institutions, should be understood as rooted in the experience of having personal rights violated. This study focuses on individual trauma, but similar findings can be seen in the disaster literature. Studying trust in governmental institutions after an earthquake disaster in Japan, for instance, Hommerich (2012) found an association between higher affectedness by the disaster and lower institutional trust. At the same time, people’s experiences with different institutions appear to impact their trust in these institutions (Hudson, 2006), and institutional failure to prevent potentially traumatic events from occurring or to adequately address such events can leave those affected with a perception of institutional betrayal, which has previously been found to exacerbate anxiety and posttraumatic reactions (Smith and Freyd, 2013).

Finally, institutional trust appears to be related to individual mental health and wellbeing. Previous studies have found a significant association between low political trust and poor psychological health, also when controlling for generalized trust (Lindström and Mohseni, 2009). Furthermore, a positive association has been found between institutional trust and perceived life satisfaction (Hudson, 2006). Other studies have also found negative associations between social capital, including trust, and negative mental health outcomes following disasters, such as PTSD, anxiety, and depression (Wind et al., 2011). Associations between low institutional trust and a lower quality of life, mental health problems, lack of social support, and barriers to seeking support from others have also been found among disaster victims (Thoresen et al., 2018).

As demonstrated by the above-mentioned studies, institutional trust may not only be of importance for upholding well-functioning democracies, but may also be a question of psychological wellbeing. It has been established that terrorist attacks can influence short-term institutional trust in the general population (Wollebæk et al., 2012; Dinesen and Jæger, 2013), but, to our knowledge, there are no previous studies that have compared post-terror institutional trust among the general population and those directly affected by the terrorist attack. Survivors from other types of disasters have been found to have lower institutional trust than the general population (Thoresen et al., 2018). Most research conducted in this field is, however, cross-sectional, and thereby unable to describe how institutional trust develops in the different phases post-disaster. The longitudinal design of the current study allowed us to investigate how institutional trust develops over time among directly affected victims. There is also a need to further investigate whether the level of institutional trust may affect health and wellbeing among those directly affected. In the current study, we investigated these issues in two separate samples, one consisting of young terror victims and the other of their parents, in two study waves at 4–5 months post-terror and 14–15 months post-terror. Among survivors, the age range was 14–57, with 90% being below 25 at the time of the interview in both study waves. The following research questions were investigated:

1. Is there a difference in institutional trust between directly affected groups after a terrorist attack and the general population?
2. How will institutional trust among directly affected populations develop over time?
3. Is institutional trust related to mental health and wellbeing in directly affected populations?

## Materials and Methods

### Research Context: The Utøya Island Terrorist Attack

On July 22, 2011, a terrorist with extreme, right-wing sympathies, intending to target the Labour Party, detonated a bomb in the government buildings in Oslo, Norway, killing eight people and injuring several more. He then traveled to Utøya, an island in a rural area approximately 40 km outside Oslo, where the Norwegian Labour Youth were gathered for their annual summer camp. Dressed as a police officer, the perpetrator pursued camp participants, shooting at those he came across. The police were notified a few minutes into the attack, and started mobilizing the local police force. However, due to several factors, including decreased staff at the local police station and lack of access to the police helicopter, mobilization took time. The perpetrator attempted to turn himself in by calling the local police approximately 40 min into the attack, however, the call was interrupted before the police managed to gather sufficient information (Nou 2012:14, 2012). Because of the severity of the situation, the police’s Emergency Response Unit (Delta) was mobilized from Oslo. However, they were also somewhat delayed due to inadequate communication with the local police regarding where to meet up and launch a police boat in order to reach the island. The police boat turned out to be too small for the heavily armed police force, and civilian boatmen had to assist in their transportation (Nou 2012:14, 2012). Members of the Emergency Response Unit reached the island and arrested the perpetrator approximately 1 h and 20 min after the shooting began (Nou 2012:14, 2012). Of the 564 individuals on the island during the attack, 69 were killed and several others were severely injured (Dyb et al., 2014; Bugge et al., 2015). Many survivors fled the island by swimming long distances in cold water to get ashore, or were rescued by civilians with boats, including individuals living in the area and tourists camping nearby.

After the attack, 495 investigative interviews with survivors were conducted by the police, most of them within the first month (Langballe and Schultz, 2017). The interviews followed an interview manual developed by the National Criminal Investigation Service (NCIS), and were conducted by members of the regional police force. Langballe and Schultz (2017) found that 72.6% of survivors did not find the interrogative interview stressful, or only to a very small extent, whereas 10.3% found it to be very stressful (p. 65). The remaining 17.1% found the interview to be somewhat stressful.

The court case against the terrorist began on April 16, 2012 and lasted 10 weeks. Prior to trial, the perpetrator had admitted to committing the acts, but had pleaded not guilty. A central question before the court case was whether the perpetrator would be declared sane or insane. This question was also important for many victims, as declaring the perpetrator insane would imply that he should be committed to psychiatric care, rather than detention. Before the trial, two expert reports written by two separate pairs of psychiatrists came to opposing conclusions regarding this question. This discrepancy sparked a comprehensive debate in the media. A verdict was announced on August 24, 2012; the perpetrator was declared sane and sentenced to preventive detention for acts of terror, premediated murder and attempted murder (Oslo District Court, 2012). During the trial, efforts were made to facilitate the victims’ presence. This included the appointment of assistance attorneys for survivors and the bereaved, and video transmission of the court case to premises in other parts of the country so that victims not residing in Oslo could follow the trial if they chose to.

In the aftermath of the terrorist attack, a commission was summoned to investigate the work of the police and other relevant public institutions. In the commission’s final report, the police action on July 22, 2011 was criticized (Nou 2012:14, 2012). For instance, a witness had observed the perpetrator acting suspiciously near the government buildings just after the initial attack and recorded his car’s registration plate. Some of the information from this witness was eventually distributed to other police districts, but it was revealed that none of the nearby police districts actually registered the information in the initial phase (Nou 2012:14, 2012). Following the release of this report, the criticism was also thoroughly debated in Norwegian media, in particular why the perpetrator had not been stopped before the attacks took place as well as why it took the police so long to mobilize the operation at Utøya island (Sethne, 2017). The police also released a report on their internal evaluation (National Police Directorate, 2012). The police’s own evaluation differed from that of the commission in several ways, most importantly when it came to whether the attack could have been prevented (Sethne, 2017).

### Participants and Procedure

Police records were used to identify survivors from the Utøya shooting. All 495 survivors were invited to partake in the study through postal invitation, with the exception of four survivors who were 13 years old or younger and one survivor living abroad, who was therefore unavailable for interviews. Survivors 13 years and younger were not included because of their age and because they were there with camp employees who had brought their smaller children to stay with them during the camp. These small children were not camp participants, and were not invited to the study. The current study uses data from wave one (4–5 months post terror) and wave two (14–15 months post-terror) of the Utøya study. In wave one, 325 survivors participated in the study, whereas 285 participated in wave two. Based on contact information collected from the survivors, their parents or caregivers were also invited to participate in the study, with the exception of parents of survivors aged 33 or older. This cut-off was set because the older survivors were not camp participants, but rather working, volunteering or visiting the camp (Haga, 2019). The parents of 482 survivors were eligible for participation (Stene and Dyb, 2016). Parents of young disaster victims are an important, but understudied, group of directly affected individuals, as experiencing your child being in danger of death or serious injury has been found to affect mental health (Thoresen et al., 2016). Among the parents and caregivers, 463 participated in wave one, whereas 435 participated in wave two. All survivors, and parents of youths born in 1992 or later, were interviewed face-to-face by trained personnel. Parents of youths born in 1991 or earlier were invited to respond to a questionnaire. The different data collection methods were utilized in order to ensure that everyone could participate, given the logistical and practical constraints of the research project. In their invitation to participate, study participants were given information about the background and purpose of the study and what participation would involve for them, as well as about how information about them would be securely stored and what voluntary participation entails. Postal information letters were sent simultaneously to survivors and their care-givers. If survivors were below 16 years of age, it was described in the letters that parental consent was necessary for participation, in accordance with Norwegian law. The study was approved by the Norwegian Regional Committee for Medical and Health Research Ethics and is based on written consent from all participants, with parents giving consent for children below the age of 16.

### Measures

Institutional trust was measured using two items (1) “How much do you trust the police?”, and (2) “How much do you trust the legal system?” Both items are scored on a scale from 0 (no trust at all) to 10 (complete trust), and are identical to items used in the European Social Survey. The levels of institutional trust among survivors and their parents were compared to levels of institutional trust in the general population, using data from the 2012 wave of the European Social Survey (ESS) (ESS Round 6: European Social Survey Round 6 Data, 2012). These data were collected throughout 2012 and 2013, with fieldwork in Norway between August 2012 and February 2013 (ESS Round 6: European Social Survey, 2018). The ESS is conducted every 2 years, surveying attitudes, beliefs and behavior in a cross-national sample, and is nationally representative for citizens above the age of 15 in over 30 European countries. Thus, the survey allows for cross-national comparison, as well as comparison over time. Data from the ESS is available on their webpage, as is information about survey methods (European Social Survey, n.d.). For this study, only ESS data from the Norwegian version of the survey was used.

Mental health and wellbeing after the terrorist attack was measured using three measures. Psychological distress was measured using the shortened, eight-item version (SCL-8) of the Hopkins Symptom Checklist-25 (Derogatis et al., 1974; Wilhelmsen, 2009; Solberg et al., 2011). This scale measures depression and anxiety symptoms in the preceding 2 weeks, on a scale from 1 (not at all bothered) to 4 (very much bothered). Six valid items were needed to compute the mean score. Cronbach’s alphas for the scale were 0.86 (wave one) and 0.90 (wave two) in the survivor sample, 0.90 (wave one) and 0.92 (wave two) in the parent sample. Post-traumatic stress reactions in the past month were measured using the UCLA PTSD Reaction Index (UCLA PTSD-RI) (Steinberg et al., 2004). The scale covers 20 items measuring post-traumatic stress (PTS) reactions. Fourteen PTS reactions are measured by single items on the scale, whereas three PTS reactions are covered by two items each. The reaction scale score thus consists of 17 items measuring PTSD according to the DSM-IV criteria on a five-point Likert scale from 0 (never) to 4 (all the time). Mean score was used for analysis, and 13 valid items were required to compute the score. Cronbach alphas were 0.89 for both wave one and wave two in the survivor sample, and 0.92 for both wave one and wave two in the parent sample. In addition, perceived quality of life was measured using one item in which participants were asked to indicate how satisfied they were with life in general on a scale from 1 (very dissatisfied) to 10 (very satisfied) (In accordance with Wilhelmsen, 2009).

Demographic information included gender, age, and ethnic background (non-Norwegian origin was defined in accordance with Statistics Norway (2018) as a person born abroad to non-Norwegian parents).

### Analyses

All parent respondents were included in the analysis when comparing with the ESS. However, only survivors aged 15 or older at the time of the interview were included in the comparison due to the lack of comparable data for younger respondents. This left us with 319 survivors from wave one and 284 survivors from wave two who were eligible for comparison. The 2012 wave of the ESS contained 1154 respondents in the comparable age group for survivors (15–57 years of age), and 1198 respondents in the comparable age group for parents (28–74 years of age).

Differences between the ESS population and the survivor and parent populations were analyzed using linear regressions. One unadjusted model and one model adjusting for age, gender, and ethnic background were estimated. In accordance with ESS recommendations, design weights and post-stratification weights were applied in the analysis. Design weights are used in the ESS to correct for sampling bias created by the fact that some individuals in a given country may not be as likely to be included in the sample due to the sampling design used there. For the sample used in this analysis, however, all design weights were = 1. Post-stratification weights are used in order to correct for sampling errors and non-response errors.

Pearson’s *r* was used for correlations between institutional trust and psychological health measures. Bootstrapping was used when calculating the confidence intervals (bias-corrected and accelerated, BC_a_, based on 10000 bootstrap replications).

IBM SPSS Statistics for Windows, version 25, and R (The R Foundations for Statistical Computing, Vienna, Austria) were used for the analysis, with the R package boot for bootstrapping.

## Results

Of the 325 survivors who participated in wave one of the study, 47.1% were women; the corresponding number was 47.0% among the 285 who participated in wave two. In wave one, 12.5% had non-Norwegian ethnic background; the corresponding number in wave two was 10.0%. Among the 453 parents who participated in wave one, 56.7% were mothers, compared to 59.7% of the 426 individuals who participated in wave two. Among the parents, 7.3% at wave one and 6.1% at wave two were ethnically non-Norwegian. In the first wave of interviews, survivors were, on average, 19.7 years old on the day of the interview (SD: 4.6), whereas parents were, on average, 48.4 (SD: 6.5) years old. At wave two, the average ages were 20.6 years (SD: 4.3) and 49.4 (SD: 6.0) years, respectively.

Average trust levels in survivors and their parents are presented in Table 1.

**TABLE 1:** Trust levels for survivors and parents range 0–10.

	**Wave 1**		**Wave 2**	
		
	**Trust in police Mean (SD)**	**Trust in legal system Mean (SD)**	**Trust in police Mean (SD)**	**Trust in legal system Mean (SD)**
Survivors	7.70 (2.40)	8.18 (2.12)	6.85 (2.70)	8.45 (2.10)
Parents	7.39 (2.24)	7.85 (2.21)	6.09 (2.61)	8.47 (1.82)

### Post-terror Institutional Trust: Comparing Survivors and Their Parents With the General Population

For the comparison with data from the European Social Survey, only survivors aged 15 or older were included in the analysis. 319 survivors from wave one (47.6% female), and 284 survivors from wave two (47.2% female) were eligible for comparison with the ESS. Average age was 19.3 years (SD: 4.6) at wave one and 20.1 years (SD: 4.2) at wave two. As there were very minor differences between the unadjusted and adjusted models when comparing the directly affected groups to the general population, only the adjusted models are presented in the table.

As displayed in Table 2, survivors had a significantly higher level of trust in the justice system compared to the general population at wave one, 4–5 months post terror. This increased further at wave two, 14–15 months after the terrorist attack. Regarding the police, however, the trend was different. At wave one the survivors had more trust in the police than the general population. At wave two, however, the survivors’ level of trust was lower than among the general population.

**TABLE 2:** Unweighted and weighted models for trust in the police and justice system for survivors and parents wave 1 (4–5 months post-terror) and wave 2 (14–15 months post-terror), compared to the general population sample (ESS).

	**Survivors**			**Parents**		
		
	**Regression coefficient**	**95% Cl**	***p*-value**	**Regression coefficient**	**95% CI**	***p*-value**
**Wave 1**						
Trust in the police (range 0–10)						
Unweighted	0.53	0.23,0.83	0.001	0.21	−0.01,0.42	0.058
Design and post-stratification weights	0.50	0.20,0.81	0.001	0.24	0.03,0.46	0.029
Trust in the justice system (range 0–10)						
Unweighted	1.13	0.83,1.42	<0.001	0.55	0.34,0.77	<0.001
Design and post-stratification weights	1.13	0.83,1.42	<0.001	0.63	0.42,0.85	<0.001
**Wave 2**						
Trust in the police (range 0–10)						
Unweighted	–0.37	−0.69,−0.05	0.022	–1.04	−1.27,−0.80	<0.001
Design and post-stratification weights	–0.38	−0.70,−0.06	0.019	–1.00	−1.24,−0.76	<0.001
Trust in the justice system (range 0–10)						
Unweighted	1.38	1.08,1.68	<0.001	1.19	0.98,1.41	<0.001
Design and post-stratification weights	1.38	1.08,1.68	<0.001	1.28	1.06,1.49	<0.001

*The table presents the effect of being in the directly affected group. The model is adjusted for age, gender, and ethnic background.*

Parents, on the other hand, did not vary significantly from the general population in terms of their trust in the police at wave one, but were significantly lower at wave two. Their trust in the justice system was higher than the general population at wave one, although not as elevated as survivors. Parents’ trust in the justice system also increased compared to the general population at wave two.

### Institutional Trust and Mental Health

As is evident in Table 3, trust in the police and the justice system were both negatively associated with post-traumatic stress symptoms and psychological distress among survivors. This was true in both waves. All survivors had sufficient valid items for mean scores for HSCL and PTSR to be calculated in both waves. For perceived quality of life, the association was positive for both institutions and in both waves, though with a somewhat stronger association at wave two.

**TABLE 3:** Terror survivors: Concurrent associations between institutional trust and anxiety and depression (HSCL), post-traumatic stress reactions (PTSR) and perceived quality of life at wave 1 (4–5 months post-terror) and wave 2 (14–15 months post-terror) with 95% confidence intervals.

**Wave 1**	*Trust in the police*	*Trust in the justice system*
*HSCL*	−0.30(−0.40,−0.19)	−0.20(−0.30,−0.09)
*PTSR*	−0.33(−0.43,−0.22)	−0.27(−0.37,−0.16)
*Quality of life*	0.19(0.07,0.32)	0.20(0.08,0.32)

**Wave 2**	*Trust in the police*	*Trust in the justice system*

*HSCL*	−0.33(−0.44,−0.21)	−0.29(−0.43,−0.17)
*PTSR*	−0.32(−0.43,−0.21)	−0.29(−0.41,−0.17)
*Quality of life*	0.30(0.16,0.43)	0.36(0.22,0.49)

*The table presents correlation coefficient for Pearson *r*-correlations. 95% confidence intervals calculated using BC_*a*_, based on 10000 bootstrap replications.*

For parents, the only significant association at wave one was found between psychological distress and trust in the justice system, and this was a weak, negative association, as can be seen in Table 4. At wave two, however, stronger, though still moderate, negative associations were found between both post-traumatic stress symptoms and psychological distress, and trust in both institutions. At wave one, *n* = 4 parents had insufficient valid items for mean score to be calculated for HSCL. The corresponding number for PTSR was *n* = 2. At wave two, *n* = 2 was missing for the HSCL score, whereas *n* = 1 was missing for PTSR. For parents, the association between institutional trust and perceived quality of life was positive in both waves, although the association was stronger at wave two.

**TABLE 4:** Parents: Concurrent associations between institutional trust and anxiety and depression (HSCL), post-traumatic stress reactions (PTSR) and perceived quality of life at wave 1 (4–5 months post-terror) and wave 2 (14–15 months post-terror) with 95% confidence intervals.

**Wave 1**	*Trust in the police*	*Trust in the justice system*
*HSCL*	−0.09(−0.19,0.01)	−0.11(−0.23,−0.01)
*PTSR*	−0.09(−0.19,0.01)	−0.10(−0.21,0.01)
*Quality of life*	0.16(0.05,0.26)	0.19(0.07,0.30)

**Wave 2**	*Trust in the police*	*Trust in the justice system*

*HSCL*	−0.24(−0.33,−0.14)	−0.15(−0.26,−0.05)
*PTSR*	−0.21(−0.30,−0.11)	−0.14(−0.24,−0.04)
*Quality of life*	0.26(0.16,0.38)	0.25(0.15,0.35)

*The table presents correlation coefficient for Pearson *r*-correlations. 95% confidence intervals calculated using BC_*a*_, based on 10000 bootstrap replications.*

## Discussion

A comprehensive literature exists on human adaptation after disasters. More knowledge is needed, however, about how directly affected individuals relate to public institutions after a disaster and the current study is, to our knowledge, the first to compare levels of trust among terror victims, to those of the general population. In an early time perspective, institutional trust among both survivors and parents was comparable to, or higher than, in the general population. One year later, however, we observed changes in institutional trust in the groups directly affected by terror. Trust in the legal system increased over time, both in survivors and their parents, but in both groups trust in the police dropped to a level significantly lower than in the general population.

In accordance with other studies (Hommerich, 2012; Thoresen et al., 2018) our results indicate that disasters can be followed by a change in levels of trust among those directly affected. However, contrary to previous studies, we found that being directly affected does not necessarily imply a negative development in levels of trust. Rather, the development turned out to be institution specific. Previous studies have found conflicting results regarding whether changes in institutional trust after experiencing disaster or other adversities appear to spread across different institutions. Whereas Hudson (2006) found that loss of trust after adverse experiences would generalize across institutions, Dinesen and Jæger (2013) found the development of institutional trust after a terrorist attack to be institution specific, and not generalizable across institutions, in accordance with our results. The two referenced studies vary, however, with regards to the type of experiences included in the analysis, as well as time passed since these adverse experiences. How the potential generalization across institutions will develop over an even longer time span than the one covered in the current study, thus remains an open question. Since most studies looking into institutional trust are cross-sectional, changes in trust over time are generally an under-theorized aspect. Thoresen et al. (2018) found that survivors from a ferry disaster had lower trust than the general population across institutions 26 years after the event. Seen in association with our results, this could suggest that the passing of time may be a key to understanding whether changes in institutional trust will generalize across institutions. As our results demonstrated, institutional trust will not necessarily decrease abruptly after experiencing a disaster. However, if distrust persists this could potentially lead to a generalization of this lack of trust across institutions. The passing of time, as well as the timing of when trust is measured in relation to events following a disaster, such as investigations and trials could therefore be highly important for understanding levels of institutional trust. Future research should incorporate time as an axis when investigating institutional trust post-terror and after other disasters, as it appears to be an important factor in understanding the development of this phenomenon.

As our results clearly illustrate, trust levels will not necessarily remain constant with the passing of time after a disaster, but can both increase and decrease over time. Thus, the current study lends support to the notion that institutional trust may develop over a lifetime, based on, for instance, institutional performance (Hudson, 2006) and the individuals’ experiences with the insititutions in question. With regards to the current study it is therefore important to consider how institutional performance, as well as people’s perceptions of institutional performance, may have developed in the time after the terrorist attacks. Measuring institutional performance objectively is challenging and beyond the scope of the current study. However, previous studies have pointed to some examples of institutional performance in the aftermath of the July 22, terrorist attacks which can help explain the development of institutional trust in this particular case. In a previous study investigating the same group of survivors, more than 7 out of 10 reported that they found the interrogative interview with the police after the terrorist attack to be not at all stressful, or only to a very small extent (Langballe and Schultz, 2017). However, the police force’s actions on the day of the terrorist attack were heavily criticized by the July 22, commission approximately a year after the attack – a criticism that was also covered extensively in Norwegian media (Nou 2012:14, 2012). There was no equivalent criticism of the legal system. On the contrary, a study found that, overall, those who testified in the court case were satisfied with how they were included in the trial and taken care of by their legal representatives (Laugerud and Langballe, 2017). The fact that the trial ended with the perpetrator being sentenced to preventive detention (Oslo District Court, 2012) may also have contributed to the sense that justice had been done.

Negative, although moderate, associations between institutional trust and post-traumatic stress reactions, anxiety, and depression were found in survivors at both waves of data collection. A similarly clear association was not observed for the parents shortly after the terrorist attack, with the exception of a negative association found between psychological distress and trust in the legal system. For this group, however, associations similar to those found in survivors could be established later on. Similarly, positive associations were found between institutional trust and perceived life quality for both survivors and parents at both waves. These findings are in line with previous literature (Wind et al., 2011; Hommerich, 2012; Thoresen et al., 2018), and emphasize the potential for negative consequences associated with low institutional trust. Studying victims of sexual assault, Smith and Freyd (2013) found that institutional betrayal, that is, the perception of being betrayed by institutions that have not upheld their responsibilities post-trauma, can worsen trauma reactions. The *perception* of institutions and their performance in the time after disasters, therefore seems to be significant in the link between institutional trust and psychological health and wellbeing. Similarly, previous studies have suggested that stress in disaster victims may, at least to some extent, result from negative experiences with public institutions (Freudenburg, 1997; Bos et al., 2005). Thoresen et al. (2018) argue that reaching out to public institutions for help after trauma, but experiencing that help is lacking or insufficient, could contribute to maintaining mental health issues over time by giving disaster victims the feeling that they are unprotected. Conversely, given that the current study found that trust will not necessarily decrease after a disaster, it highlights a potential for positive development post-disaster.

### Strengths and Limitations

The current study is, to our knowledge, the first to measure institutional trust longitudinally among a group of individuals directly affected by a terrorist attack. Strengths include the longitudinal design, the ability to compare levels of institutional trust with data from the general population, the inclusion of two separate samples, the relatively high response rate and relatively low attrition rate, and the low level of missing data. There are some limitations that should be taken into consideration. First of all, every disaster is different, occurs in a specific place and context, and to a particular group of victims. The groups studied in this paper are unique in some respects, which could limit the generalizability of the results. In the Utøya case, the survivors, as well as their parents, can be assumed to represent individuals with a high level of political affinity, given that the survivors were present at a summer camp organized by the Labour Youth, which is presumably associated with particularly high institutional trust (Catterberg and Moreno, 2005). It should also be noted that the two groups studied are not independent of each other, and that one could expect that survivors’ levels of institutional trust could be affected by their parents’ levels, and vice versa. Further, the study design enabled the examination of correlations, but could not establish causality. Thus, one cannot exclude the possibility that other underlying variables are involved. Additionally, as with other longitudinal studies, non-response and attrition may have affected study results. Previous analyses of the survivor sample, found that those who only participated in the first wave of interviews, were more likely to be of non-Norwegian origin, to report higher terror exposure, and not being members of the Labour Youth, as compared to those who participated in both waves (Stene and Dyb, 2016). At the same time, those who only participated in the second wave of interviews, were more likely to report higher levels of psychological and somatic symptoms, compared to those who participated in both rounds. Given that the study found an association between post-disaster negative mental health outcomes and lower levels of institutional trust, a potential implication could be overestimation of institutional trust in both study waves. We did not account for potential clustering in the parent sample. An additional shortcoming of the study was the one-item measure of trust in the police and the justice system, respectively. There is an extensive literature on the measurement of trust, demonstrating that it is a complex and multidimensional construct (e.g., Moorman et al., 2018; Ruelens et al., 2018). In the present study, we were unable to engage in more complex or extensive measurement of trust, because of the need to keep questionnaires short for the particular population sampled, as well as the need to compare constructs measured in this sample with those measured in the ESS. It is important that future studies measure trust more comprehensively, to explore these phenomena in more detail. Finally, the first measurement of institutional trust among survivors was made 4–5 months after the terrorist attack, but the only comparison material which could be found was gathered 13–19 months after the attack. However, institutional trust has been shown to be stable over time in the Norwegian general population (Catterberg and Moreno, 2005).

## Conclusion

From the current study we can conclude that directly affected groups’ institutional trust appears to differ from that of the general population following terrorist attacks, although direct trauma experiences do not necessarily imply weakened institutional trust. Rather, this study suggests an institution specific development of trust, which could imply that institutional performance and the victims’ experiences with particular institutions are significant for determining trust levels. Furthermore, the passing of time after a disaster and the unfolding of events in the aftermath, such as investigations and trials, may influence trust levels, including whether and when they will generalize across institutions.

Lower institutional trust is associated with psychological distress and could therefore affect victims negatively if it persists. However, given that institutional trust appears to be institution specific, and thereby reliant on how these institutions appear and are perceived, findings from the current study highlight the potential institutions have for nurturing a healing environment post-terror.

### Further Research

First and foremost, there is a need for studies of institutional trust integrating time as an axis in their study design, enabling further scrutiny of the importance of the passing of time for the development of institutional trust and the generalization of trust across institutions. Further, there is a need for studies measuring perceptions of institutional performance in more detail and how this is related to institutional trust. Studies examining the processes involved when victims of terror are in contact with public institutions, and how this affects their institutional trust, would also be beneficial. Finally, there is a need for further studies comparing different groups of directly affected individuals to increase our understanding of how institutional trust may vary across groups, contexts, and different types of disasters.

## Data Availability Statement

The datasets used to analyze terror survivors and their parents for this manuscript are not publicly available because they contain identifiable and sensitive data. Requests to access the datasets should be directed to GD, grete.dyb@nkvts.no. The data used to analyze the general population in this study was obtained from a third party, the European Social Survey. These data can be used without restrictions, for not-for-profit purposes. For further information on conditions of use, please refer to www. europeansocialsurvey.org/data/conditions_of_use.html. The datasets can be accessed through www.europeansocialsurvey.org.

## Ethics Statement

The Utøya study, involving terror survivors and their parents, was reviewed and approved by the Norwegian Regional Committee for Medical and Health Research Ethics (Approval Nos. REK HSø 2011/1625 and REK N 2014/246). Written informed consent to participate in this study was provided by all participants, with the participants’ legal guardian/next of kin giving consent for children below the age of 16.

## Author Contributions

GD is the PI of the study and responsible for the data collection. GD and ST contributed to data collection. All authors contributed to the conception and design of the study and revisions of the manuscript, and read and approved the submitted version. LN and TW-L performed the statistical analysis. LN wrote the first draft of the manuscript. ST wrote sections of the manuscript.

## Conflict of Interest

The authors declare that the research was conducted in the absence of any commercial or financial relationships that could be construed as a potential conflict of interest.
